# Does retirement reduce familiarity with Information and Communication Technology?

**DOI:** 10.1007/s11150-021-09573-8

**Published:** 2021-07-03

**Authors:** Danilo Cavapozzi, Chiara Dal Bianco

**Affiliations:** 1 Department of Economics, Ca’ Foscari University of Venice, Fondamenta San Giobbe, Cannaregio 873, 30121 Venezia, Italy; 2 Department of Economics and Management, University of Padua, Via Del Santo 33, 35123 Padova, Italy

**Keywords:** Computer skills, Internet, Retirement, Instrumental variables., J14, J21, J24.

## Abstract

This paper analyses the effect of retirement on the familiarity with Information and Communication Technology (ICT) of older individuals. We argue that inability to cope with ICT might represent a threat for older individuals’ social inclusion. To account for the potential endogeneity of retirement with respect to familiarity with ICT, we instrument retirement decision with the age-eligibility for early and statutory retirement pension schemes. Using data from the Survey of Health, Ageing and Retirement in Europe, we show that retirement reduces the computer literacy and the frequency of internet utilization for men and women. This finding is robust to the inclusion as control factors of health, cognition and social network indicators, which the literature has shown to be affected by retirement. Overall, the reduction in the familiarity with ICT after retirement tends to be stronger in the long-run.

## Introduction

The use of Information and Communication Technology (ICT) is a major issue for older individuals. Based on data drawn from the 2003 wave of the Eurobarometer, Peacock & Künemund (2007) show that the percentages of computer users and internet users among individuals aged 55–64 amount only to 35 and 26% respectively and fall to 11 and 8% when considering those aged 75 or over. A decade later, OECD (2017) shows that in the OECD countries the percentage of individuals aged 55–74 using internet reaches 63%, but in all countries the percentage of internet users in this age range is lower than the overall percentage in the country. Although the access to ICT of older individuals shows a positive trend, they are still at risk of falling behind the rest of the population.

Employment is expected to be one of the main drivers of the diffusion of ICT among older individuals due to the pervasive and always increasing presence of ICT in job organization and in the definition of job contents. Nevertheless, older workers approach the end of their working careers. The exit from labour market might reduce their proficiency and utilization of ICT and widen their gap in this respect with younger individuals. In this paper we investigate the effect of retirement on computer literacy and internet use of older individuals using data from the Survey of Health, Ageing and Retirement in Europe (SHARE).

Inability to cope with ICT might represent a threat for older individuals’ social inclusion. Seifert et al. (2020) argue that during the COVID-19 pandemic older adults have been less likely than younger individuals to use digital services to reduce the isolation risks due to physical distancing. According to recent data on individuals aged 50 or over living in Europe during the pandemic (Wave 8 COVID-19 of SHARE), the probability of experiencing depression, trouble with sleep and loneliness is significantly lower by about 20% for those who in previous waves of the survey declare to have good computer skills and use internet at least weekly.^1^ Likewise, their probability of daily contacts with other people not living in the household by phone, email or any other electronic means is significantly higher by about 15%. ICT proves to be important to preserve social ties when safety measures prevent individuals from having physical contacts. Lack of access to ICT might boost social isolation and inequalities since it is likely to be more prevalent for individuals with a more disadvantaged background. Gell et al. (2015) find that in the US older adults with health problems are less likely to use technological devices. Analogous conclusions are drawn by König et al. (2018) for Europe. Using the German Ageing Survey, Huxhold et al. (2020) show that the probability of having internet access is lower for older individuals with lower education, income and cognitive abilities. Yu & Fiebig (2020) based on the China Health and Retirement Longitudinal Study confirm that individuals with higher cognitive abilities are more likely to be internet users and also report that, conditional on baseline scores for cognitive functioning, internet users experience a milder cognitive decline, thus suggesting a protecting role of internet access to preserve cognitive functioning late in life. Analogously, Almeida et al. (2012) use Australian data and find that individuals who use computers present a lower risk of dementia.

The ways in which ICT enters daily life are manifold. ICT is of help to organize leisure activities, including travelling or going to museums or the theatre (Näsi et al., 2012), or to deal with the increasing digitalization of services. Webster (2020) documents a worldwide increase in the provision of health care virtual treatments to avoid physical meetings during the COVID-19 pandemic. Bogan (2008) uses the Health and Retirement Study (HRS) and finds that the probability of owning stocks exhibits a stronger increase over time for households using computers (interpreted as a proxy for internet use), who face lower information and transaction costs. ICT also makes it easier to communicate with relatives and social network members by e-mail, video-call and chat applications (Wellman, 2001). Huxhold et al. (2020) find that individuals in the oldest age group in their sample (70–85 years old) who provide informal care to their grandchildren have higher probabilities of having internet access. Everything else constant, familiarity with ICT is expected to improve individuals’ well-being by supporting the strength of their social ties and providing a manifold support to the activities in which they are, or would like to be, engaged.

We analyze the effect of retirement on computer literacy and internet use by drawing data from the waves 5 and 6 of SHARE. Our sample includes individuals aged 50–69 and living in thirteen European countries plus Israel. Whereas computer literacy is measured by respondents’ self-evaluations, the definition of internet use is based on the answers to a summary question asking respondents whether they have used internet at least once in the last seven days. We interpret these outcomes as two alternative measures of the concept of familiarity with ICT. The former measure focuses on proficiency, the latter on the frequency of utilization.

Retirement is a major life-course event. A wide literature, which we will briefly review in the next section, has already studied the effects of retirement on several domains relevant for the social inclusion of older individuals, such as cognitive functioning, health and social networks. Our paper contributes to this literature by investigating the effect of the exit from the labour force on the proficiency and frequency of utilization of ICT among older individuals. Employment might guarantee a continuous access to ICT facilities. Gaining at work a higher familiarity with word-processing, spreadsheet elaborations, emailing, video-calling, web-browsing and search engines can have immediate spin-offs on the various ways in which individuals can avail themselves of ICT in their non-labour market activities. Nevertheless, the sign of the effect of retirement on the familiarity with ICT is a priori ambiguous. On the one hand, individuals who retire have no longer available the opportunities provided by their jobs to use ICT as well as to maintain and develop their knowledge of ICT, making their skills more at risk of fading away or becoming obsolete. On the other hand, the exit from the labour market might expand the amount of time available for leisure activities and this might act as an incentive to utilize ICT devices by exploiting their current knowledge and improving it through learning-by-doing or interactions with social network members. Which of these two opposite effects prevails is an empirical issue.

Analyzing the causal effect of retirement on the familiarity with ICT is complicated by endogeneity concerns. Typically unobserved individuals’ characteristics are likely to affect both retirement decisions and skills in managing ICT. For instance, ambition and dedication might make workers more willing to invest in their ICT knowledge in order to increase their productivity and improve their job prospects. As long as these skills are rewarded in the labour market, individuals with higher familiarity with ICT devices might also find attractive to delay their exit from the labour force (Biagi et al., 2013; Friedberg, 2003), bringing about reverse causality concerns. Moreover, individuals who have a higher marginal utility of leisure might have found outside the labour market the occasions and the incentives to invest in their ICT knowledge, for instance in order to organize their leisure time. Everything else constant, these individuals might be familiar with ICT devices regardless of their job tasks and would like to retire as soon as possible to expand the amount of time devoted to their non-labour market activities.

We address the endogeneity of retirement using an instrumental variable approach. Following the literature analyzing the effect of retirement on several well-being domains that will be discussed in the next section, our instruments are based on institutional information concerning the eligibility ages for early and statutory retirement, which vary across countries, genders and birth cohorts, as well as over time.

The main findings of our analysis show that retirement significantly reduces computer skills and internet utilization for both men and women. This pattern remains confirmed even after controlling for measures of other well-being domains affected by retirement, such as health, cognitive abilities and social networks size. We also carry out a heterogeneity analysis of the retirement effect by splitting the sample by job characteristics, education levels, marital status and presence of children. Finally, we inspect the time-dynamics of the effect of interest and find that after three years since retirement retirees are associated with a reduction in computer skills and web utilization as compared with workers. However, we also find that in the first year after retirement the frequency of web use of men increases.

The paper continues as follows. Section 2 surveys the literature investigating the effect of retirement on several well-being dimensions. Section 3 presents the data. Section 4 describes the estimation strategy. Section 5 discusses our findings. Section 6 concludes.

## Literature review: the consequences of retirement

Our paper relates to the literature focusing on the consequences of retirement from work on non-monetary well-being dimensions such as cognitive functioning, physical and mental health, health behaviours and social interactions. The papers in this field typically share endogeneity concerns of retirement with respect to the outcome of interest mainly due to omitted variable bias and reverse causality. As an example, individuals with worse levels of cognition might be those more likely to retire since they might experience a higher disutility of work and face worse job career expectations. This source of endogeneity is usually addressed by instrumenting the retirement indicators using cross-country and time variation in the Social Security system rules produced by the pension reforms of the last decades.^2^

One of the first dimensions investigated in this literature has been cognitive functioning. The epidemiological and gerontological literature outlined that cognitive abilities, such as memory, verbal fluency and numeracy, decline as individuals age. It becomes important to assess the contribution to this pattern provided by major life-time events associated with ageing, of which retirement is a key example. Rohwedder & Willis (2010) assemble an international cross-sectional dataset based on HRS, the English Longitudinal Study of Ageing (ELSA) and SHARE and find a negative effect of retirement on cognition. Bonsang et al. (2012) use the longitudinal dimension of HRS and confirm a negative impact of retirement on cognitive functioning. They stress that the pension reforms extending the permanence of older workers in the labour market might have positive externalities for their cognitive abilities. In line with this evidence, Mazzonna & Peracchi (2012), draw data from SHARE and find that after retirement there is an increase in the rate of cognitive decline. Cognitive functioning is also found to significantly vary across education groups and European regions. Still based on SHARE, Celidoni et al. (2017) show that the effect of retirement depends on the timing of the exit from the labour force. Whereas it is negative in the long run for individuals retiring at the statutory eligibility age, it becomes positive in the short run and negligible in the long run for those retiring as soon as they become eligible for an early retirement option. Atalay et al. (2019) use the Household, Income and Labour Dynamics in Australia (HILDA) and document that the short-run negative effect of retirement on the cognitive levels of older Australians is smaller for women than for men and an analogous cross-gender difference is found when looking at the effect of retirement duration. They also document that after retirement women become more likely to be engaged in reading, club memberships, household and volunteer activities, whereas this is not the case for men. A more stimulating environment reduces the negative consequences of retirement on cognition.

Coe & Zamarro (2011) investigate the effect of retirement on health based on SHARE data. They find that retirement improves individuals’ health status, which is alternatively measured by respondents’ health self-evaluations, proved to be a significant predictor of mortality, and a composite index combining more objective health indicators based on respondents’ answers to specific questions on their health condition. Gorry & Slavov (2021) use ELSA to estimate the consequences of retirement on a set of health biomarkers. They found mixed evidence and in many cases the retirement impact is not statistically significant. These findings are not in contrast with the evidence in Coe & Zamarro (2011). As the authors argue, health biomarkers estimates are less exposed to measurement error and biases typical of self-evaluations but focus on specific aspects of individuals’ health. Retirement might instead improve health aspects that are not described by the biomarkers typically considered in a survey and captured by self-evaluations. The health consequences of retirement can be heterogeneous across the health indicators considered as well as subgroups in the population. Based on SHARE, Godard (2016) shows that retirement does not produce any significant change in women weight but instead increases the probability of being obese for men, in particular if they have an already high obesity risk and retire from strenuous jobs. Consistently, Mazzonna & Peracchi (2017) draw data from SHARE and show that the effect of retirement on health and cognitive abilities varies across occupational groups. Although the exit from the labour force typically has an overall negative effect that magnifies as the number of years spent in retirement increases, it turns out to have an immediate positive effect that remains significant after 10 years for individuals previously employed in more physically demanding jobs. Heller-Sahlgren (2017) uses SHARE data and documents that the effect of retirement on mental health is negligible in the short run but negative in the long run, suggesting a protective role of mental health preservation played by pension reforms postponing retirement. Belloni et al. (2016) combine four waves of SHARE covering the period 2004–2013 and find that retirement improves the mental health of individuals, in particular former blue-collar workers, in periods and regions that are more severely hit by the economic crisis. Kolodziej & García-Gómez (2019) based on SHARE data assess whether the retirement effect on mental health varies over the mental health distribution. They show that although, on average, mental health improves after retirement, these gains are stronger for individuals just below or above the threshold used to identify the occurrence of depression in the Euro-D scale. A positive effect of retirement on physical and mental health is also found by Eibich (2015) using the German household survey GSOEP. His investigation of the factors underlying this relation suggests it might be explained by an increase in sleep duration on weekdays, a more active lifestyle and the absence of job related stress and strain. The literature is devoting an increasing attention to the identification of the potential mechanisms underlying the effect of retirement on health. Celidoni & Rebba (2017) use SHARE and find that retirement reduces the probability of being inactive or not doing any vigorous physical activity. This effect is stronger for individuals with high education and with a more affluent parental background. Based on the same survey, Celidoni et al. (2020) show that retirement increases daily fruit and vegetable consumption for men but not for women.

Finally, retirement might have consequences on the size and composition of individuals’ social networks. Borsch-Supan & Schuth (2014) estimate the causal effect of retirement on social networks in SHARE and show that the exit from the labour force decreases the size of social networks and changes their composition by reducing the proportion of nonfamily members. Consistently, Patacchini & Engelhardt (2016) use the US longitudinal survey National Social Life, Health, and Aging Project and find that retirement reduces the size and density of social networks. These effects are driven by women and individuals with high education. Comi et al. (2020) exploit the longitudinal dimension of SHARE and document that, in line with Borsch-Supan & Schuth (2014), retirement changes the composition of social networks by increasing the proportion of family members and decreasing the proportion of colleagues and friends. However, they do not detect any significant variation in the social network size.

Our paper analyses the effect of retirement from work on the familiarity with ICT. The connection with the literature surveyed in this section is twofold. First, addressing this research question is important per se to develop a more complete picture of the consequences of retirement on older individuals’ social inclusion, given the support provided by ICT in organizing individuals’ activities and fostering their active participation in the society. Second, cognitive functioning, health and social networks are dimensions impacted by the retirement process. These dimensions might also affect the incentives and the costs to access ICT and maintain adequate skills to manage ICT devices. As outlined in the Introduction, individuals with worse cognitive functioning and health status might be more limited in using ICT. Those with more dense social networks might find a stronger incentive to use ICT to maintain their social ties. We will enrich the set of covariates in our baseline specifications to include indicators of these dimensions in order to assess the robustness of our findings.

## Data

We use the waves 5 and 6 of SHARE, which have been collected in 2013 and 2015 respectively. SHARE is a multidisciplinary and multi-country survey whose population of reference consists of individuals aged 50 or over and their spouses living in Europe and Israel. Data are collected by CAPI questionnaires that are ex-ante standardized and allow meaningful cross-country comparisons of respondents’ answers. SHARE interview gathers information on several dimensions relevant for individual well-being, including employment, health, social and family networks, income and wealth.

In our sample we include individuals who self-classify themselves as either at work or retired from work.^3^ We consider only individuals who are currently at work as employees or whose last job was as employees. We decide to exclude current and former self-employed individuals because they might formally retire but remain partially involved in their previously-run business (for instance, a family business), blurring the distinction between retirement and employment.^4^ SHARE is a longitudinal survey. To establish consistency over time for the retirement definition, we classify respondents as retired starting from the first wave they self-declare to be in this state.^5^ The questionnaires administered in the interviews conducted in waves 5 and 6 of SHARE elicit respondents’ computer literacy and internet use by means of two questions. The first question asks to rate computer skills by answering to the question “*How would you rate your computer skills?*” according to the predetermined scale “*1. Excellent, 2. Very good, 3. Good, 4. Fair, 5. Poor, 6. I never used a computer*”.^6^ The second question focuses on the use of internet and asks “*During the past 7 days, have you used the Internet, for e-mailing, searching for information, making purchases, or for any other purpose at least once?*” (Yes/No).^7^

These two questions allow describing two different aspects of the familiarity with ICT of older individuals. The former question provides us with a self-assessment of the level of computer literacy, which includes, but it is not confined to, browsing the web since it also reflects skills with other computer applications and with managing hardware issues. Based on this question, we derive a binary indicator that takes on value 1 when individuals have at least good computer skills and zero otherwise (*Computer skills*). The latter question instead provides a summary measure of the frequency of utilization of internet in the last seven days. We therefore generate a binary measure that takes on value 1 if the individual reports to have used internet in the last week (*Using internet*). Considering these two measures outlines to what extent older individuals are successful in managing ICT devices and how much frequently they use these skills.

Our sample consists of 41,869 observations referring to 26,923 individuals aged 50–69 and living in Denmark, Sweden, Belgium, Luxembourg, France, Germany, Austria, Switzerland, Spain, Italy, Estonia, Czech Republic, Slovenia and Israel. There is a longitudinal component in our sample since about one half of respondents (14,897) have been interviewed in both waves. All our analyses will be conducted separately by gender. The sample for men includes 19,188 observations (12,512 individuals), the sample for women includes 22,681 observations (14,411 individuals).

**Fig. 1:**
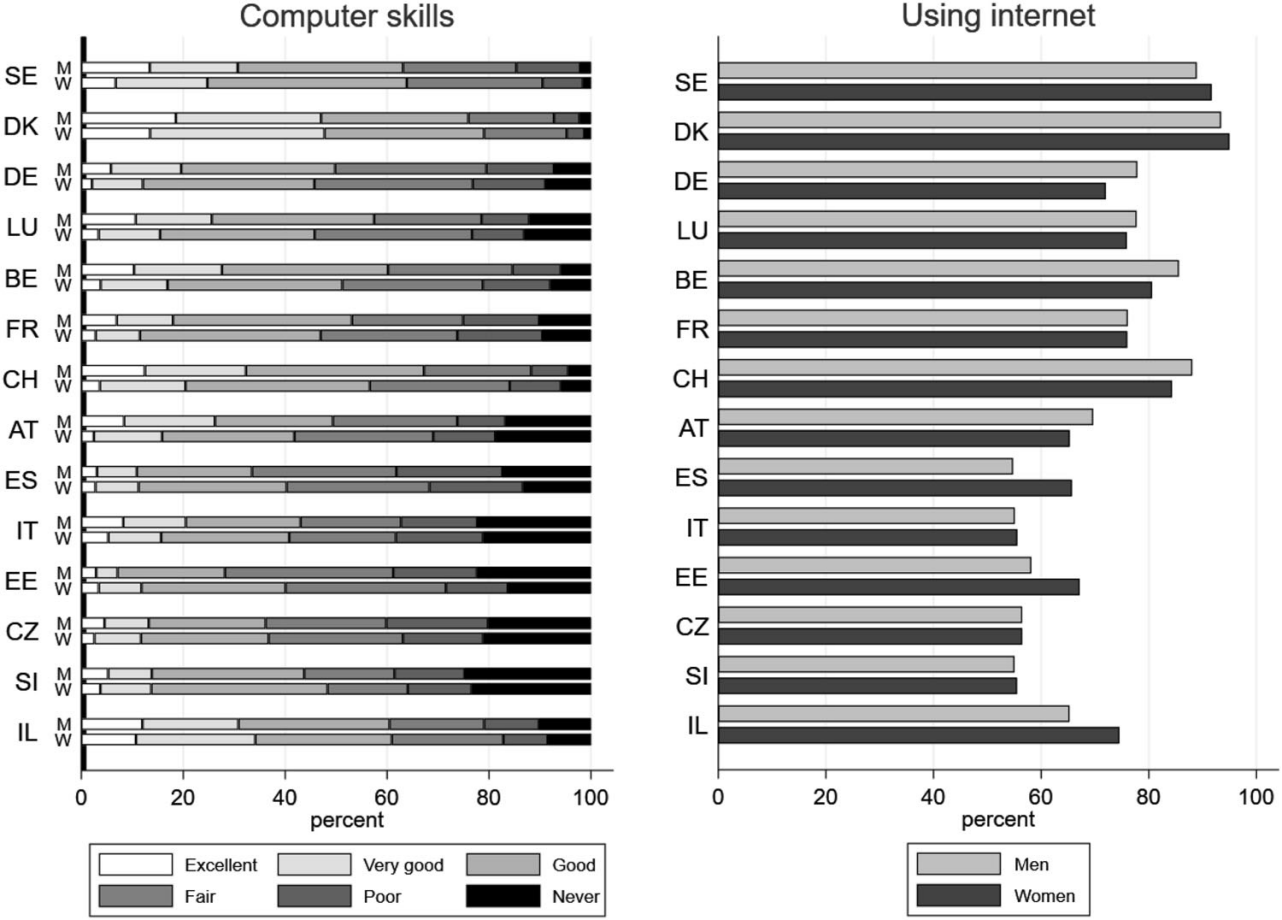
Computer skills (left panel) and percentage of individuals who have used internet in the last seven days (right panel), by country and gender

Overall, 50% of respondents in our sample think they have at least good computer skills and 72% have browsed the web in the last week (see Table 1). Figure 1 shows that for both genders there is a sizeable North-South gradient in the familiarity with ICT of older Europeans. For instance, in Denmark the percentage of men who rate their computer skills as at least good is 76%, whereas it is 33% in Spain. If we look at the other countries, we see that computer literacy is higher in Switzerland and Sweden and lower in Estonia and Czech Republic. Similar patterns arise for women. As for internet use, 72% of the men in our sample have used internet at least once in the last week. This percentage shrinks to less than 60% in Spain, Italy, Czech Republic, Slovenia and Estonia, whereas it jumps to more than 85% in Denmark, Sweden, Switzerland and Belgium. These empirical findings align with the evidence presented in OECD (2017). Web users among individuals aged 50 or over are overall more widespread in Northern Europe than in Mediterranean countries, mimicking the pattern arising from computer skills.

**Table 1:** Sample averages of the variables used in the analysis, by gender and employment status

	Men			Women		
Variable	All	Workers	Retired	All	Workers	Retired
Computer skills	0.51	0.60	0.40	0.49	0.60	0.36
Using internet	0.72	0.82	0.61	0.72	0.84	0.58
Eligible_ER	0.56	0.24	0.92	0.58	0.25	0.96
Eligible_SR	0.35	0.05	0.69	0.40	0.06	0.81
Couple	0.85	0.85	0.85	0.73	0.76	0.70
Lower/upper secondary educ.	0.45	0.46	0.44	0.45	0.44	0.45
Tertiary educ.	0.29	0.34	0.24	0.30	0.37	0.23
Age	60.8	57.1	65.0	60.2	56.4	64.7
Poor health	0.04	0.02	0.07	0.04	0.03	0.07
Adl	0.08	0.04	0.13	0.08	0.05	0.11
Iadl	0.09	0.04	0.15	0.12	0.07	0.19
Blue collar	0.37	0.34	0.41	0.22	0.17	0.27
Public sector	0.31	0.28	0.34	0.44	0.43	0.45
Children	0.90	0.90	0.90	0.92	0.91	0.92
Grandchildren	0.56	0.42	0.71	0.63	0.50	0.78
Wave 6	0.44	0.43	0.45	0.45	0.45	0.45
Observations	19,188	10,055	9,133	22,681	12,319	10,362

In the sample, 48% of men and 46% of women are retired. The fractions of individuals reporting good computer skills (*Computer skills*) or having used internet (*Using internet*) by retirement status provide prima facie evidence of the negative correlation between retirement and familiarity with ICT. As reported in Table 1, on average, 60% of male workers have at least good computer literacy, whereas only 40% of male retirees reach this level of skills. This difference is confirmed when looking at women. In all the countries considered, men and women at work declare to have higher computer skills than retirees. This evidence is clearly descriptive as differences between workers and retirees are partly attributable to other individual characteristics (such as age), that we will control for in the following analyses.

We exploit contextual information concerning Social Security systems to determine whether an individual meets the age requirements set by early retirement (ER) and statutory retirement (SR) schemes. Whereas for current employees we consider the age requirements in place at the time of the interview, for retirees we consider those in place at the reported retirement year. Almost all retirees satisfy the age requirement for an Early Retirement (*Eligible_ER*) scheme (92% for men and 96% for women), whereas 69% of male retirees and 81% of female retirees are age eligible for a Statutory Retirement (*Eligible_SR*) pension. On the contrary, the fractions of workers eligible to ER or SR schemes are much lower. Overall, only one fourth of workers meet the age requirement for the ER route and less than 10% do so for SR.

In the sample, 45% of men have lower or upper secondary education and 29% have tertiary education. They are on average 60.8 years old, most of them live with a partner (85%), 90% have children and 56% have grandchildren. Women are less likely to live with a partner (73%) and to work (or to have worked) as blue collars (22 versus 37%) than men, but they are more likely to work in the public sector (44 versus 31%). Among both men and women, about 4% declare to be in poor health. Consistently, the average number of limitations with (instrumental) activities of daily living (*(i)adl*) is remarkably low for both men and women as most of the individuals in our sample do not suffer from these problems.^8^ For both genders, retirees are 8 to 9 years older than workers, less healthy and more likely to have worked as blue collars and in the private sector.

## Empirical strategy

To address the effect of retirement on the familiarity with ICT, we estimate the following linear probability model:

1 $$y = \beta _0 + \beta _1retired + {\boldsymbol{\beta}} _2^\prime {\boldsymbol{x}} + u$$

where *y* is the outcome variable considered, *retired* is the key explanatory variable, **x** is a vector of individual and household characteristics and *u* is the error term. As mentioned above, we run all our econometric specifications separately by gender. To account for the presence of the longitudinal component in our sample, in all our regression models standard errors are clustered to account for arbitrary correlation in the error term between observations referring to the same individual.

The set of controls in the vector *x* includes country of residence fixed effects, a second order polynomial in age, a time fixed effect to discriminate between observations of the waves 5 and 6 of SHARE, education (*lower/upper secondary* and *tertiary education*; having less than secondary education is the baseline group), current health (*poor health*), number of limitations with activities of daily living (*adl*), number of limitations with the instrumental activities of daily living (*iadl*), a set of controls for the family structure (*couple*, *children* and *grandchildren*) and country-specific quartile dummies for household net wealth (*hh wealth second/third/fourth quartile*). Moreover, we control for job characteristics. For current employees this information refers to their current job, for retirees it refers to their last job before retirement (*blue collar* and *public sector*).

Ordinary least square (OLS) estimation of Eq. (1) provides a consistent estimate of the causal effect of retirement on the familiarity with ICT (*β*_1_) only if retirement is not correlated with the error term *u*.

However, the unobserved component *u* summarizes personal traits, such as ambition and dedication, which might affect both the timing of retirement and the propensity to maintain up-to-date ICT knowledge. For instance, individuals with a stronger labour market attachment and a stronger desire to meet career and job promotion goals might be more likely to delay retirement and more likely to invest in their human capital, which includes the proficiency in computer and web utilization, in order to keep their knowledge up-to-date and reap the rewards offered by the labour market for these skills. This reasoning also suggests the presence of a reverse causality issue: in a labour market characterized by continuous technological innovations, individuals who are more prone to invest in their ICT knowledge are also more likely to face the working conditions that make retirement postponement more attractive. As long as retirement lowers the familiarity with ICT, failing to control for this source of heterogeneity might lead to a spurious amplification of this negative effect.

Moreover, the error component *u* includes individual heterogeneity in the marginal utility of leisure. Individuals who place a higher importance on their leisure time activities might have developed better ICT knowledge to organize them and might have a stronger desire to retire as soon as possible to expand the amount of leisure time available. Neglecting this dimension of heterogeneity across individuals would lead to an attenuation in the negative effect of retirement emerging at the descriptive level.

We solve this threat to the identification of the causal effect of retirement on the familiarity with ICT by an instrumental variable strategy. We rely on two dummy variables *Eligible*_*ER* and *Eligible*_*SR* indicating whether respondents are eligible to early and statutory retirement schemes respectively. As discussed in the previous section, these variables are defined by combining individual-level information for country of residence, gender and birth-cohort with country-specific institutional information reflecting the minimum age requirements needed to access the early and statutory retirement routes. See Online Appendix B for details on the eligibility rules.

Figure C1 and C2 in Online Appendix C report the proportion of men and women who are retired as a function of the years since/to eligibility age, for statutory and early retirement respectively. These figures show that there are sizable increases in the proportion of retired at both statutory and early retirement age (we also report the same figures separately for each country, Figures C3- C6).

Using institutional information concerning pension systems makes us confident that our instruments are not under the direct control of the individuals and are arguably exogenous (Angelini et al., 2009; Battistin et al., 2009; Rohwedder & Willis, 2010).

## Results and discussion

### OLS and IV results

In Table 2 we report OLS estimates of Eq. (1) using as outcomes our two binary measures of familiarity with ICT in turn.

**Table 2:** Effect of retirement on the probability of having at least good computer skills and having used internet in the last seven days

	Men		Women	
	(1)	(2)	(3)	(4)
Variables	Computer skills	Using internet	Computer skills	Internet
Retired	−0.049*** (0.011)	−0.047*** (0.010)	−0.071*** (0.011)	−0.067*** (0.010)
Observations	19,188	19,188	22,681	22,681

Linear probability models estimated by OLS

Additional controls: couple, education, age, age squared, poor health, adl, iadl, household wealth quartiles, blue collar, public sector, have children, have grandchildren, time dummy, country dummies. Standard errors clustered at the individual level. The full set of estimation results is in Table A2 in Online Appendix A

***p < 0.01; **p < 0.05; *p < 0.1

The first two columns refer to men. In the first column we consider the probability of having at least good computer skills. The coefficient on the dummy variable *retired* is negative and statistically significant. It shows that, conditional on the observable covariates in the **x** vector, the probability of having good computer literacy reduces by 4.9 percentage points for those who are retired. This variation is sizeable as it accounts for about 10% of the sample average for this outcome. The second column reports the estimates when considering the probability of having used internet in the last week. Retirees are 4.7 percentage points less likely than employees to have used internet in the last seven days. On average, the proportion of web users in our population of interest decreases by 7%. As before, this variation is statistically significant.

As for the other control variables, the full set of results in Table A2 shows that the probabilities of having good computer literacy and browsing the web at least on a weekly basis are significantly higher for individuals with higher education and higher wealth levels, who live in a couple and are healthier. Cross-country differentials remain substantial even after conditioning on the right-hand-side variables in our specifications. Columns 3 and 4 summarize the results of the same analysis conducted for women. Likewise men, female retirees are significantly less likely to have good computer skills (−7.1 percentage points) and less likely to use internet frequently (−6.7 percentage points).

The OLS results neglect any endogeneity issue of retirement with respect to familiarity with ICT. In particular, the error term *u* is assumed to be uncorrelated with retirement decisions, although we argued in the previous sections that both reverse causality and unobserved heterogeneity cast doubts on this assumption. In the reminder of the paper, we relax this assumption and estimate the model in Eq. (1) using instrumental variable techniques and relying on the exogenous variability induced by heterogeneity in eligibility rules for early and statutory retirement schemes (*Eligible_ER* and *Eligible_SR*).

Table 3 reports the Two-stage Least Square (2SLS) estimates. For both genders, our previous OLS findings are confirmed by the instrumental variable estimation and the magnitude of the point estimates increases.^9^

**Table 3:** Effect of retirement on the probability of having at least good computer skills and having used internet in the last seven days

	Men		Women	
	(1)	(2)	(3)	(4)
Variables	Computer skills	Using internet	Computer skills	Using internet
Retired	−0.104** (0.044)	−0.079** (0.038)	−0.092*** (0.030)	−0.070** (0.030)
Observations	19,188	19,188	22,681	22,681
Sargan-Hansen p-value	0.513	0.794	0.187	0.097
Eligible_ER	0.241*** (0.025)	0.241*** (0.025)	0.215*** (0.027)	0.215*** (0.027)
Eligible_SR	0.209*** (0.024)	0.209*** (0.024)	0.370*** (0.031)	0.370*** (0.031)
Weak identification	132.141	132.141	217.888	217.888

Linear probability models estimated by 2SLS

Additional controls: couple, education, age, age squared, poor health, adl, iadl, household wealth quartiles, blue collar, public sector, have children, have grandchildren, time dummy, country dummies. Standard errors are clustered by country and cohort. Stock-Yogo weak identification test critical values: 10% maximal IV size 19.93, 15% maximal IV size 11.59, 20% maximal IV size 8.75, 25% maximal IV size 7.25. The full set of estimation results is in Table A3 in Online Appendix A

***p < 0.01; **p < 0.05; *p < 0.1

At the bottom of Columns 1 of Table 3 we report first-stage coefficients for the two instruments used: *Eligible_ER* and *Eligible_SR* prove to be strongly correlated with retirement decisions. Everything else constant, those who are eligible for early or statutory retirement schemes are significantly (at the 1% level) more likely to be retired. The F-statistic of the joint significance of the instrumental variables in the first-stage equation takes a value well above the Stock & Yogo (2005)’s critical values (which are reported below the table) suggesting that instruments are not weak.

Given that our model is over-identified - we have two additional instruments for one endogenous regressor – we can compute the Sargan-Hansen over-identification test. This test allows to check whether our instruments are uncorrelated with the error term *u*. The p-values of the Sargan-Hansen tests show that for men the null hypothesis of exogeneity of the instruments is not rejected. Instead, for women this hypothesis receives a weaker support (p-values are much lower) and it is rejected at the 10% level in the case of web utilization.

This result is unexpected given that our instruments, which are widely used in the literature to account for the endogeneity of retirement, mainly depend on two dimensions that are arguably exogenous: the age of respondents at the time of the interview and the eligibility rules of the pension schemes for early and statutory retirement.

However, as discussed in Angrist & Pischke (2009), the rejection of the null hypothesis of the Sargan-Hansen test might be due to heterogeneity in the effects identified by two exogenous instruments rather than an endogeneity problem. Our two instruments are indeed associated with two different complier groups. Eligibility to early retirement schemes defines a group of compliers consisting of individuals who retire as soon as they qualify for the early retirement option (*early retirees*). Eligibility to statutory retirement schemes instead defines a group of compliers who find the exit from the labour market less attractive and retire only once they become eligible to the statutory retirement scheme (*statutory retirees*). To assess whether the retirement effect is heterogeneous across complier groups, we re-run our instrumental variable estimation for women using the two additional instruments one at a time and show the results in Table 4.

**Table 4:** Effect of retirement on the probability of having at least good computer skills and having used internet in the last seven days

	Early retirees		Statutory retirees	
	(1)	(2)	(3)	(4)
Variables	Computer skills	Using internet	Computer skills	Using internet
Retired	−0.040 (0.050)	−0.012 (0.045)	−0.112*** (0.034)	−0.094*** (0.034)
Observations	22,681	22,681	22,681	22,681
Eligible_ER	0.265*** (0.031)	0.265*** (0.031)		
Eligible_SR			0.400*** (0.033)	0.400*** (0.033)
Weak identification	73.501	73.501	149.103	149.103

Linear probability models estimated by 2SLS in the women sample using one instrument at a time

Additional controls: couple, education dummies, age, age squared, poor health, adl, iadl, household wealth quartiles, blue collar, public sector, have children, have grandchildren, time dummy, country dummies. Standard errors clustered at the country and year of birth level. Stock-Yogo weak identification test critical values: 10% maximal IV size 16.38, 15% maximal IV size 8.96, 20% maximal IV size 6.66, 25% maximal IV size 5.53

***p < 0.01; **p < 0.05; *p < 0.1

Columns 1 and 2 refer to the early retirees. We find that the effect of retirement on computer literacy and on the probability of using internet at least weekly is insignificant. The third and the fourth columns report estimation results for the statutory retirees. Retirement significantly reduces their probability of having good computer literacy and using the web in the last week by 11.2 percentage points and 9.4 percentage points respectively. The differences in the retirement effect across complier groups are statistically significant at the 10% level.^10^ This evidence corroborates the explanation that the weaker support for the null hypothesis of validity of the overidentifying restriction in the Sargan-Hansen test for women is dictated by heterogeneity in the retirement effects between complier groups rather than endogeneity issues of the instruments.^11^

### Potential mechanisms

The literature reviewed in Sections 1 and 2 documents that familiarity with ICT varies with health, cognitive functioning, and social networks and that retirement is a major lifetime event producing a deep impact on these dimensions, which then might act as mechanisms underlying our relationship of interest. From a policy perspective, it becomes important to assess the magnitude of the differences between retirees and employees in ICT familiarity after conditioning on dimensions potentially impacted by retirement. In this section we augment the set of control factors by including indicators for mental health, cognitive abilities and social networks.^12^ Finding that the retirement effect remains unaffected by the inclusion of these further controls would strengthen the need of implementing programs fostering the diffusion of ICT among retirees to counterbalance the genuine detrimental impact of the exit from the labour force. Conversely, if the effect of retirement shrank once conditioning on these dimensions, it would suggest that the effect of retirement on ICT familiarity goes mainly through mental health, cognitive functioning, and social networks.

The results are summarized in Table 5, which shows the 2SLS estimates of the retirement effect produced by the specifications considered.

**Table 5:** Effect of retirement on the probability of having at least good computer skills and having used internet in the last seven days

	Men		Women	
	Computer skills	Using internet	Computer skills	Using internet
*Additional controls:* Euro-d scale				
Retired	−0.099** (0.044)	−0.085** (0.038)	−0.095*** (0.030)	−0.072** (0.030)
Observations	19,036	19,036	22,527	22,527
*Additional controls:* number of words recalled, fluency test, two numeracy scores				
Retired	−0.105** (0.044)	−0.076** (0.037)	−0.085*** (0.030)	−0.061** (0.030)
Observations	18,990	18,990	22,527	22,527
*Additional controls*: social network size				
Retired	−0.159*** (0.050)	−0.123*** (0.044)	−0.092*** (0.032)	−0.058* (0.031)
Observations	13,382	13,382	16,526	16,526
*Additional controls*: number of activities				
Retired	−0.099** (0.044)	−0.073* (0.038)	−0.091*** (0.030)	−0.070** (0.030)
Observations	19,188	19,188	22,681	22,681

Linear probability models estimated by 2SLS adding additional controls

Our main specification described by Eq. (1) is estimated including the specified additional controls. Baseline controls: couple, education, age, age squared, poor health, adl, iadl, household wealth quartiles, blue collar, public sector, have children, have grandchildren, time dummy, country dummies. Standard errors are clustered by country and cohort

***p < 0.01; **p < 0.05; *p < 0.1

We first look at the role of mental health measured by the Euro-d depression scale, which aggregates in a score spanning from 1 to 12 the outcomes with respect to a battery of conditions related with mental health problems: depression, pessimism, suicidality, guilt, sleep, interest, irritability, appetite, fatigue, concentration, enjoyment and tearfulness.^13^ The retirement effect is still negative and significant after controlling for the Euro-d measure. It is also worth noting that the point estimates of the coefficients on the retirement variable across our specifications are very close to those reported in Table 3.

We also control for a battery of measures of cognitive functioning, namely memory, fluency and numeracy. Memory is measured by a recall test in which respondents are first read ten words and then asked to recall them immediately and after an interference period (roughly 5 min later). The indicator we considered takes the total number of words correctly recalled in the two trials. The fluency measure is the number of names of different animals respondents can list in one minute. Finally, we consider the scores respondents obtained in two numeracy tests asking them to solve problems involving the utilization of percentages, fractions, multiplications and subtractions. Again, the inclusion of these indicators does not qualitatively alter the magnitude and the significance of the retirement effect.

Moreover, we control for a measure of the social network size of our respondents derived from their answers to a question asking to declare the number of persons important for them. The sample size reduces since this question has been asked in wave 6 only. However, the coefficient on the retirement variable remains sizeable and statistically significant. Finally, we control for the number of activities respondents have done in the last twelve months.^14^ This can be an additional measure of their potential network connections and it can also capture incentives to use ICT associated with undertaking these activities. All our results are confirmed and the size of the retirement effect proves to be remarkably stable.

Overall, the evidence in this section supports the hypothesis that the effect of retirement on ICT familiarity is not spuriously determined by other well-being dimensions impacted by the exit from the labour force. Despite the inclusion of these controls, retirement is found to have a negative and significant effect on computer skills and frequency of web utilization.

### Heterogeneity analysis

In this section we carried out a heterogeneity analysis to inspect to what extent the relationship of interest varies across groups in the population. This exercise is important to understand whether the retirement effect found before is driven by specific population subgroups, who should then receive particular attention in the design of policy interventions. The results are summarized in Table 6, which reports 2SLS estimates and the results of a formal test for the significance at the 10% level of the difference between the retirement effects obtained for each comparison considered.^15^

**Table 6:** Effect of retirement on the probability of having at least good computer skills and having used internet in the last seven days

	Blue collar	White collar	White collar high skills	White collar low skills	Private sector	Public sector	Lower/upper sec. educ.	Tertiary educ.	Single	Couple	No children	With children
Men												
Computer skills	−0.037 (0.059)	−0.142** (0.066)	−0.182** (0.078)	−0.068 (0.102)	−0.068 (0.054)	−0.184** (0.080)	−0.047 (0.049)	−0.264*** (0.097)	−0.233* (0.135)	−0.092** (0.047)	−0.029 (0.113)	−0.111** (0.046)
Difference is significant at 10%	NO		NO		NO		YES		NO		NO	
Using internet	−0.019 (0.062)	−0.108** (0.050)	−0.103** (0.048)	−0.065 (0.091)	−0.055 (0.046)	−0.149** (0.059)	−0.081* (0.044)	−0.059 (0.058)	−0.408*** (0.128)	−0.035 (0.039)	−0.081 (0.112)	−0.080** (0.038)
Difference is significant at 10%	NO		NO		NO		NO		YES		NO	
Observations	7,135	12,053	7,552	4,501	13,273	5,915	13,628	5,560	2,838	16,350	1,929	17,259
Women												
Computer skills	−0.068 (0.043)	−0.096** (0.039)	−0.076 (0.067)	−0.090** (0.043)	−0.070* (0.038)	−0.118*** (0.046)	−0.111*** (0.032)	0.024 (0.082)	−0.061 (0.052)	−0.101*** (0.037)	0.145 (0.117)	−0.109*** (0.030)
Difference is significant at 10%	NO		NO		NO		NO		YES		YES	
Using internet	−0.02 (0.047)	−0.081** (0.037)	−0.077 (0.061)	−0.072* (0.042)	−0.075** (0.037)	−0.065 (0.040)	−0.067** (0.033)	−0.051 (0.047)	−0.083* (0.048)	−0.072* (0.037)	−0.220** (0.103)	−0.059* (0.031)
Difference is significant at 10%	NO		NO		NO		NO		NO		YES	
Observations	4,910	17,771	6,973	10,798	12,684	9,997	15,815	6,866	6,037	16,644	1,870	20,811

Linear probability models estimated by 2SLS for different subsamples

Our main specification described by Eq. (1) is estimated for several subsamples. Additional controls: couple, education, age, age squared, poor health, adl, iadl, household wealth quartiles, blue collar, public sector, have children, have grandchildren, time dummy, country dummies. Standard errors are clustered by country and cohort. For each comparison of interest, the significance of the difference between the effects found in the two groups is formally tested at the 10% significance level by a bootstrap procedure based on 500 replications of our estimates obtained in bootstrap samples stratified by wave and country of residence

***p < 0.01; **p < 0.05; *p < 0.1

The presence of ICT widely differs across jobs. There is a negative gap of 60 percentage points in the probability of using a computer at work for blue collars with respect to white collars in our sample. As a consequence, we might argue that the drop in ICT familiarity associated with retirement emerging from our results may be driven by the white collar workers, who were more substantially exposed to ICT at the workplace. The consequences of retirement on ICT knowledge might be wider for this group of workers since their exit from the labour market precludes them from exploiting the intense utilization of ICT devices and applications featuring their jobs. The first two columns of Table 6 show that the familiarity with ICT always significantly decreases with retirement for white collars, but not for blue collars; however, the differences between the retirement effects in the two groups are never statistically significant.

The white collar category is particularly broad since it includes jobs with different contents and required skills. We split the white collars in two groups depending on the characteristics of their jobs: white collars with high skills and white collars with low skills.^16^ The former group includes individuals with higher level of competencies managing jobs probably requiring a more intense and autonomous use of ICTs for professional purposes. Retirement is found to reduce both the probability of having good computer literacy and the frequency of the web utilization in all the groups of interest, but the effect is significant only among white collar men with high skills and white collar women with low skills. However, for both genders, the effect does not significantly vary between white collars with high and low skills.

Consistently, the difference between the retirement effect for public and private sector workers is never statistically significant.

The sample is also split by educational attainments given the higher probability of being computer literate and frequent web users documented by our estimates (see Tables A3 in the Appendix A). Individuals with higher education might have the skills to learn how to use computers and the internet and keep their knowledge updated more easily. With the exception of computer skills for men, we find that the retirement effect is actually negative and significant only for individuals with low education.

Turning to family characteristics, we consider separately singles and those living as a couple. The retirement effect is always negative for both groups, although in few cases the reduced sample size jeopardizes its statistical significance. In particular, we do not reject the hypothesis that the retirement effect on internet use is stronger for singles among males. This finding suggests that having a partner might be supportive of maintaining the familiarity with internet, for instance to organize common activities in leisure time (Cavapozzi and Zantomio, 2021), to maintain contacts with children or to manage joint financial investments. Conversely, for women, we found that the detrimental retirement effect on computer skills is stronger for those who live in couple, whereas the effect on internet use does not vary across groups. This pattern might be related to the difference between our measures of familiarity with ICT. Instead of focusing on the frequency of internet utilization, our computer skills indicator is expected to also capture proficiency with setting hardware devices and software products. Our results suggest that partnered women who retire seem to delegate the management of these issues to other people, for instance their spouses, and as a result they do not practice these skills.

We also split the sample by presence of children. On the one hand, having regular and frequent contacts with children is an incentive to use computers and the web that might sweep away the negative retirement effect found in the overall sample. In addition, children might provide informal training to their parents in using ICTs and web applications. On the other hand, individuals without children might have developed their ICT skills more autonomously given that they cannot build on the informal training support from the offspring. Then, they might be more effective in keeping these skills up to date even after leaving the labour force. We find that for men the retirement effect on computer skills and internet use is significant and negative for individuals with children and not significant for those without children, but the difference between the two groups is not statistically significant. For women, retirement induces a significant reduction in web use regardless of the presence of children but the effect is significantly stronger for women without children. The opposite occurs for computer skills as the negative effect is stronger for women with children. Women might delegate to children the management of the ICT devices needed to use email, video-call and chat products. This way, women with children are more likely than their counterparts without children to lose the skills to manage these tasks, which are arguably captured by our computer skills measure. At the same time, they exploit their children expertise to have these internet-based tools operational in order to stay in touch with social network members, including their children themselves.

### Time dynamics

We then investigated the time dynamics of the effect of retirement on ICT familiarity. The Introduction motivates the presence of two opposite channels determining this effect. The former suggests a negative effect induced by the fact that remaining at work constitutes a channel for older workers to keep their ICT skills updated and routinely used. The latter channel instead suggests a positive effect due to the expansion of leisure time produced by retirement, which might strengthen individuals’ incentives to invest in their ICT knowledge to organize recreational activities or manage their social networks. The timing with which these channels materialize is different. Although the expansion of leisure time after retirement is immediate, it is unlikely that, despite the continuous technological change, the ICT skills of retirees become obsolete right after retirement because they are no longer kept updated at the workplace. Which of these two opposite channels prevails in the short and in the long run is an empirical issue. In order to shed light on the presence of time dynamics in the effect of interest, we split retirees in three mutually exclusive groups depending on the amount of time spent as retirees at the time of interview: at most 1 year (13.63% of retirees in our sample), between 2 and 3 years (21.79%) and more than 3 years (64.59%). Next, we modified our estimating equations by replacing our retirement dummy with three dummies indicating these three groups.^17^

Our results are collected in Table 7 and support the hypothesis that in the long run retirement is detrimental for ICT familiarity. In all our specifications, retirees who have left the workforce for more than 3 years are less likely than workers to have good computer skills and have used the web at least once in the last week (though the parameter is not statistically significant in the first column). However, there are some differences in the time dynamics between the outcomes considered. Focusing on computer literacy, the magnitude of the retirement effect significantly increases with the time spent in retirement for women, whereas it seems to decrease for men. The findings for web utilization instead suggests that there is a positive “honeymoon effect” in the short run for men. For both genders the effect is negative and significant in the long run. This heterogeneity across outcomes might be related to the fact that job tasks in a professional environment are more usually carried out by a computer rather than a smartphone, which is instead particularly widespread for personal use. Also, web resources (including messaging and social media applications) can be exploited by using a smartphone and not necessarily a computer. As a result, immediately after retiring individuals might be more exposed to a decrease in their proficiency with computer skills rather than in the frequency of web use.

**Table 7:** Effect of retirement duration on the probability of having at least good computer skills and having used internet in the last seven days

Variable	Men		Women	
	Computer skills	Using internet	Computer skills	Using internet
At most 1 year	−0.122 (0.084)	0.126* (0.070)	−0.032 (0.059)	−0.049 (0.055)
Between 2 and 3 years	−0.053 (0.057)	−0.186*** (0.053)	−0.054 (0.042)	0.030 (0.043)
More than 3 years	−0.047 (0.062)	−0.105** (0.052)	−0.145*** (0.031)	−0.144*** (0.035)
Observations	19,187	19,187	22,679	22,679

Linear probability models estimated by 2SLS

In our main specification described by Eq. (1) we substitute the retirement dummy with a set of dummies for retirement duration. Additional controls: couple, education, age, age squared, poor health, adl, iadl, household wealth quartiles, blue collar, public sector, have children, have grandchildren, time dummy, country dummies. Standard errors are clustered by country and cohort

***p < 0.01; **p < 0.05; *p < 0.1

### Fixed-effects 2SLS estimator

We exploit the longitudinal dimension of our sample to estimate our equations of interest by Fixed-Effects (FE)-2SLS on the subsample of individuals who are interviewed in both wave 5 and 6. This leads to drop 31 and 27% of the observations for men and women respectively. The FE-2SLS estimation procedure first applies the FE transformation to remove individual-specific time invariant heterogeneity potentially correlated with all our covariates and then estimates the transformed equation by 2SLS to take into account additional time varying heterogeneity causing the endogeneity of retirement decisions.

In a FE-2SLS framework the identification of the effect of retirement rests on individuals transiting from work to retirement and becoming eligible for ER or SR over the short time period (about two years) under consideration. In our longitudinal balanced sample, only 9% (7%) of men (women) transit from work to retirement, 10% (9%) become eligible for ER and 13% (10%) for SR between waves 5 and 6.^18^ This subsample of newly retirees is unlikely to be representative of the whole population of reference in our study. In our full sample used for the main analysis retirees on average have left the labour force for 5.8 years, and 25% have retired for more than 8 years. Given the short panel at our disposal, the FE-2SLS estimator identifies the retirement effect building upon the information conveyed by newly retirees and then it could be interpreted as a short-run effect of retirement. Although we refrain from considering the FE-2SLS analysis as a robustness check of our main findings in light of these differences in the reference population, we think it could complement the findings emerging from our time dynamics analysis summarized in the previous subsection. Whereas in the previous subsection the short-run effect was defined as referring to the first year after retirement, in the FE-2SLS framework it is defined as the retirement effect identified by transitions towards retirement occurring between the two waves. On average, the time distance between the interviews conducted in the two waves amounts to two years but it varies from 14 to 33 months.

The results produced by the FE-2SLS estimator are reported in Table 8 and display similarities with the results in Table 7 when looking at individuals who have retired since at most one year. The effect of retirement on computer skills for men is negative and significant. It is worth noting that the magnitude of the coefficient is in line with the corresponding short-run effect found in Table 7. As for women, the effect is confirmed to be not statistically significant. The retirement effect on internet use for men is again positive and statistically significant, with a magnitude close to its counterpart in Table 7. Finally, it turns out to be negative and significant for women.

**Table 8:** Effect of retirement on the probability of having at least good computer skills and having used internet in the last seven days

	Men		Women	
	(1)	(2)	(3)	(4)
Variablesd	Computer skills	Using internet	Computer skills	Using internet
Retired	−0.171* (0.098)	0.155** (0.075)	−0.017 (0.095)	−0.154** (0.065)
Observations	13,320	13,320	16,474	16,474
Individuals	6,660	6,660	8,237	8,237
Sargan-Hansen p-value	0.038	0.466	0.628	0.469
Eligible_ER	0.090*** (0.022)	0.090*** (0.022)	0.037** (0.017)	0.037** (0.017)
Eligible_SR	0.172*** (0.030)	0.172*** (0.030)	0.223*** (0.029)	0.223*** (0.029)
Weak identification	25.597	25.597	36.428	36.428

Linear probability models estimated by FE-2SLS on the longitudinal sample

Additional controls: first difference of couple, age, age squared, poor health, adl, iadl, household wealth quartiles, blue collar, public sector, have children, have grandchildren. Standard errors clustered at the country and cohort level. Stock-Yogo weak identification test critical values: 10% maximal IV size 19.93, 15% maximal IV size 11.59, 20% maximal IV size 8.75, 25% maximal IV size 7.25

***p < 0.01; **p < 0.05; *p < 0.1

## Conclusions

In this paper we analyze the effect of retirement on the familiarity with ICT of older individuals in a sample of current and previous employees aged 50–69 and living in thirteen European countries plus Israel. Data are drawn from the waves 5 and 6 of SHARE. Familiarity with ICT is measured by two alternative binary indicators: having at least good computer skills and having used internet in the last seven days.

The identification of the effect of retirement is complicated by its potential endogeneity. We estimate linear probability models by 2SLS. Our instruments are based on age eligibility requirements for Social Security benefits, which vary across countries, genders, birth cohorts and over time. For both genders, retirees are shown to have a significantly lower probability of having good computer skills and having used internet in the last week. This finding is robust to the inclusion of indicators of health, cognitive functioning and social network among the control variables in our model. Controlling for these factors, which the literature has documented to be impacted by retirement, does not qualitatively affect the magnitude of the negative effect of retirement on the familiarity with ICT. We document how this effect varies when looking at particular subgroups in the population defined according to job characteristics, education and family composition. We also detect a time dynamics in the detrimental effect of retirement, which strengthens over time. Individuals who have retired for more than three years tend to have worse computer skills and lower frequency of web utilization.

The exit from the labour force is shown to reduce the computer skills and the frequency of internet utilization of older individuals. This pattern calls for initiatives that boost their digital inclusion. Examples of these initiatives might be financial incentives to buy ICT devices and internet subscriptions, to overcome the constraints of individuals living in more disadvantaged economic conditions, as well as the support to institutions, such as universities of the third age, that can organize courses to keep up to date the ICT knowledge of individuals in this age group (Cattaneo et al., 2016).

Moreover, the pension reforms implemented in the last decades that tightened the age eligibility requirements for pension benefits might have produced an externality in the production of ICT knowledge at later ages. Older individuals who would be otherwise less likely to use ICT find in the postponement of their retirement from the labour force an occasion to invest in their proficiency with computers and internet use. This knowledge can produce spillover effects in their non-labour market activities. Facilitating contacts with social network members, coping with digitalized services and supporting the organization of leisure time activities improve the well-being of older individuals and secure their social inclusion in the golden years.

## Supplementary information

Online Appendix

## Footnotes

1 Authors’ own calculations. We consider measures of familiarity with ICT observed at the time of wave 5 and wave 6 (2013 and 2015 respectively) since they can be taken as predetermined at the time of the pandemic.

2 A related literature has also investigated the effect of retirement on time use and dealt with analogous identification issues (see Atalay et al., 2020 for a recent contribution).

3 The question asks “*In general, which of the following best describes your current employment situation?*” Answering categories are the following: “*1. Retired, 2. Employed or self-employed, 3. Unemployed, 4. Permanently sick or disabled, 5. Homemaker, 97. Other*”. The questionnaire remarks that the category “*1. Retired*” refers to retired from own work only.

4 We further exclude individuals who transit to retirement from either a long period (at least two waves) of unemployment or from disability and women who retire after having declared being homemakers as prevalent economic status in previous waves. Among individuals always observed as retired, we exclude those who declare to have never worked.

5 Our results remain quantitatively the same if we re-classify as workers those retirees who are retired from work in wave 5 but declare as main economic status employed in wave 6.

6 The last answering category is selected by the interviewer only if respondent spontaneously declares she has never used a computer.

7 We do not use wave 7 since most of the respondents received a life history interview that does not include the questions needed to develop these measures (see Börsch-Supan et al., 2019 for more information about the administration of the life history interview in wave 7). The question about internet use asked in wave 4 has a different wording and it is not considered.

8 Activities of daily living include dressing, walking across a room, bathing or showering, eating, getting in and out of bed and using the toilet. Instrumental activities of daily living include using a map, preparing a hot meal, shopping for groceries, making telephone calls, taking medications, doing work around the house or garden, managing money, leaving the house independently, accessing transportation services and doing personal laundry. The last three activities are considered in wave 6 only.

9 Appendix D shows that our conclusions are confirmed when considering an alternative measure of computer literacy that takes on value 1 for individuals with at least very good computer skills and 0 otherwise. Moreover, we also use as outcome the variable in its original 6-item scale. Results available upon request show that retirement significantly reduces computer skills and confirm the evidence proposed by our main findings.

10 We carried out 500 replications of our 2SLS estimation based on bootstrap samples stratified by wave and country of residence and formally tested the significance of the difference between the retirement effects obtained for the two complier groups by following a bootstrap procedure. Results are available upon request.

11 We carried out the same analysis for men but, in line with the Sargan-Hansen results, we do not find any statistically significant difference in the retirement effects across complier groups.

12 We already control for self-reported health and for two indicators of physical health (adl and iadl). Excluding these variables from our baseline specification does not affect our results.

13 Descriptive statistics for the Euro-d depression scale and the other variables used in this subsection are provided in Table A1 of the Online Appendix A.

14 These activities are chosen among a list including: done voluntary or charity work, attended an educational or training course, gone to a sport, social or other kind of club, taken part in a political or community-related organization, read books, magazines or newspapers, did word or number games such as crossword puzzles or Sudoku, played cards or games such as chess.

15 Likewise before, we carried out 500 replications of our estimates based on bootstrap samples stratified by wave and country of residence and formally tested the significance at the 10% significance level of the difference between the retirement effects obtained for the two groups of interest by following a bootstrap procedure.

16 The group of white collar with high skills consists of legislators, senior officials, managers, professionals, technicians and associate professionals. The group of white collar with low skills includes clerks, service workers, shop workers and market sales workers.

17 The instruments are two sets of dummies indicating whether the individuals are eligible for early and statutory retirement since 1 year, from 2 to 3 years and since more than 3 years, for a total of 6 instruments.

18 The within variability of the retirement dummy and the instruments is always less than one third of their between variability.
