# Supplementary material for: Does retirement reduce familiarity with Information and Communication Technology?
Source: Rev Econ Househ. 2021 Jul 3;20(2):553–77. doi: 10.1007/s11150-021-09573-8 (PMC8254456; doi:10.1007/s11150-021-09573-8)
Supplement: Supplementary file 1 — Online Appendix [file 11150_2021_9573_MOESM1_ESM.docx]

Online Appendix to: “Does retirement reduce familiarity with Information and Communication Technology?”

Danilo Cavapozzi and Chiara Dal Bianco

**Appendix A: Full set of tables for descriptive statistics and main estimation results**

Table A1: Sample averages of the variables used in the analysis of potential mechanisms (subsection 5.2).

|  | Men | | | Women | | |
| --- | --- | --- | --- | --- | --- | --- |
| Variable | All | Workers | Retired | All | Workers | Retired |
| Euro-d scale | 1.62 | 1.54 | 1.71 | 2.32 | 2.24 | 2.41 |
| Number of words recalled | 10.10 | 10.66 | 9.47 | 11.17 | 11.73 | 10.50 |
| Verbal fluency | 22.79 | 23.88 | 21.60 | 23.80 | 24.59 | 22.86 |
| Numeracy | 3.83 | 3.93 | 3.72 | 3.59 | 3.65 | 3.52 |
| Numeracy 2 | 4.54 | 4.61 | 4.48 | 4.48 | 4.53 | 4.42 |
| Social network size | 2.43 | 2.48 | 2.38 | 2.95 | 3.09 | 2.81 |
| Number of activities | 2.25 | 2.33 | 2.16 | 2.50 | 2.57 | 2.41 |

Note: the number of words recalled (memory test) ranges from 0 to 20. The two numeracy tests range from 0 to 5 – the higher the score the higher is the number of questions correctly answered. The range for social network size and the number of activities is 0-7.

Table A2: Effect of retirement on the probability of having at least good computer skills and having used internet in the last seven days. Linear probability models estimated by OLS.

|  | Men | | Women | |
| --- | --- | --- | --- | --- |
|  | (1) | (2) | (3) | (4) |
| VARIABLES | Computer skills | Using internet | Computer skills | Using internet |
|  |  |  |  |  |
| retired | -0.049*** | -0.047*** | -0.071*** | -0.067*** |
|  | (0.011) | (0.010) | (0.011) | (0.010) |
| couple | 0.037*** | 0.056*** | -0.025*** | 0.011 |
|  | (0.011) | (0.010) | (0.008) | (0.007) |
| lower/upper secondary educ. | 0.112*** | 0.133*** | 0.129*** | 0.134*** |
|  | (0.010) | (0.010) | (0.009) | (0.010) |
| tertiary educ. | 0.284*** | 0.226*** | 0.212*** | 0.220*** |
|  | (0.012) | (0.011) | (0.011) | (0.010) |
| age/10 | 0.299* | 0.398*** | 0.634*** | 0.641*** |
|  | (0.157) | (0.138) | (0.142) | (0.124) |
| age squared/100 | -0.034** | -0.043*** | -0.062*** | -0.063*** |
|  | (0.013) | (0.012) | (0.012) | (0.010) |
| poor health | -0.080*** | -0.086*** | -0.058*** | -0.087*** |
|  | (0.016) | (0.017) | (0.015) | (0.016) |
| adl | -0.013 | -0.000 | 0.010 | -0.001 |
|  | (0.009) | (0.009) | (0.008) | (0.008) |
| iadl | -0.015** | -0.025*** | -0.047*** | -0.040*** |
|  | (0.007) | (0.008) | (0.007) | (0.007) |
| blue collar | -0.254*** | -0.212*** | -0.253*** | -0.240*** |
|  | (0.009) | (0.009) | (0.009) | (0.010) |
| public sector | -0.010 | -0.000 | -0.005 | 0.018*** |
|  | (0.008) | (0.007) | (0.007) | (0.006) |
| children | 0.023* | 0.044*** | -0.033** | 0.022** |
|  | (0.013) | (0.012) | (0.013) | (0.011) |
| grandchildren | -0.029*** | -0.003 | -0.015* | -0.026*** |
|  | (0.009) | (0.007) | (0.008) | (0.007) |
| Hh wealth 2nd quartile | 0.022** | 0.057*** | 0.020** | 0.022** |
|  | (0.010) | (0.010) | (0.010) | (0.009) |
| Hh wealth third quartile | 0.044*** | 0.097*** | 0.056*** | 0.054*** |
|  | (0.010) | (0.010) | (0.010) | (0.009) |
| Hh wealth fourth quartile | 0.079*** | 0.126*** | 0.083*** | 0.091*** |
|  | (0.011) | (0.010) | (0.010) | (0.009) |
| Wave 6 | 0.004 | 0.049*** | 0.010** | 0.063*** |
|  | (0.005) | (0.004) | (0.005) | (0.004) |
| AT | 0.056*** | -0.030* | 0.036* | 0.019 |
|  | (0.020) | (0.017) | (0.019) | (0.017) |
| SE | 0.182*** | 0.159*** | 0.204*** | 0.217*** |
|  | (0.019) | (0.014) | (0.018) | (0.013) |
| ES | -0.003 | -0.077*** | 0.061*** | 0.055*** |
|  | (0.017) | (0.016) | (0.018) | (0.017) |
| IT | 0.069*** | -0.103*** | 0.044** | -0.072*** |
|  | (0.018) | (0.017) | (0.019) | (0.017) |
| FR | 0.095*** | 0.033** | 0.065*** | 0.098*** |
|  | (0.019) | (0.016) | (0.019) | (0.016) |
| DK | 0.229*** | 0.128*** | 0.296*** | 0.185*** |
|  | (0.017) | (0.013) | (0.016) | (0.013) |
| CH | 0.177*** | 0.104*** | 0.114*** | 0.133*** |
|  | (0.020) | (0.015) | (0.021) | (0.016) |
| BE | 0.114*** | 0.094*** | 0.073*** | 0.104*** |
|  | (0.017) | (0.013) | (0.018) | (0.014) |
| CZ | 0.030* | -0.076*** | 0.075*** | 0.013 |
|  | (0.017) | (0.016) | (0.016) | (0.015) |
| SL | 0.044** | -0.150*** | 0.135*** | -0.068*** |
|  | (0.019) | (0.018) | (0.017) | (0.017) |
| ET | -0.079*** | -0.085*** | 0.030* | 0.040*** |
|  | (0.017) | (0.017) | (0.016) | (0.014) |
| IL | 0.095*** | -0.127*** | 0.163*** | 0.038 |
|  | (0.031) | (0.028) | (0.028) | (0.024) |
| LU | 0.140*** | 0.058*** | 0.074*** | 0.116*** |
|  | (0.022) | (0.018) | (0.026) | (0.022) |
|  |  |  |  |  |
| Observations | 19,188 | 19,188 | 22,681 | 22,681 |

Note: Standard errors clustered at the individual level. *** p<0.01, ** p<0.05, * p<0.1.

Table A3: Probability of having at least good computer skills and having used internet in the last seven days. Linear probability models estimated by 2SLS.

|  | Men | | Women | | |
| --- | --- | --- | --- | --- | --- |
|  | (1) | (2) | (3) | | (4) |
| VARIABLES | Computer skills | Using internet | Computer skills | | Using internet |
|  |  |  |  |  | |
| retired | -0.104** | -0.079** | -0.092*** | -0.070** | |
|  | (0.044) | (0.038) | (0.030) | (0.030) | |
| couple | 0.038*** | 0.056*** | -0.025*** | 0.011 | |
|  | (0.011) | (0.011) | (0.008) | (0.008) | |
| lower/upper secondary educ. | 0.111*** | 0.133*** | 0.128*** | 0.134*** | |
|  | (0.011) | (0.012) | (0.010) | (0.010) | |
| tertiary educ. | 0.280*** | 0.224*** | 0.211*** | 0.220*** | |
|  | (0.012) | (0.013) | (0.014) | (0.013) | |
| age/10 | 0.213 | 0.347** | 0.604*** | 0.636*** | |
|  | (0.187) | (0.168) | (0.155) | (0.167) | |
| age squared/100 | -0.023 | -0.037** | -0.058*** | -0.063*** | |
|  | (0.016) | (0.015) | (0.013) | (0.015) | |
| poor health | -0.074*** | -0.083*** | -0.057*** | -0.087*** | |
|  | (0.016) | (0.017) | (0.017) | (0.016) | |
| adl | -0.012 | 0.000 | 0.010 | -0.001 | |
|  | (0.008) | (0.009) | (0.008) | (0.009) | |
| iadl | -0.014** | -0.025*** | -0.046*** | -0.040*** | |
|  | (0.007) | (0.008) | (0.007) | (0.006) | |
| blue collar | -0.253*** | -0.211*** | -0.253*** | -0.240*** | |
|  | (0.010) | (0.010) | (0.009) | (0.011) | |
| public sector | -0.008 | 0.000 | -0.004 | 0.018*** | |
|  | (0.009) | (0.007) | (0.008) | (0.007) | |
| children | 0.020 | 0.043*** | -0.033** | 0.022* | |
|  | (0.013) | (0.012) | (0.013) | (0.011) | |
| grandchildren | -0.027*** | -0.002 | -0.015* | -0.026*** | |
|  | (0.009) | (0.008) | (0.008) | (0.008) | |
| Hh wealth 2nd quartile | 0.022** | 0.057*** | 0.020** | 0.022** | |
|  | (0.010) | (0.011) | (0.009) | (0.009) | |
| Hh wealth third quartile | 0.045*** | 0.097*** | 0.056*** | 0.054*** | |
|  | (0.010) | (0.011) | (0.009) | (0.009) | |
| Hh wealth fourth quartile | 0.079*** | 0.126*** | 0.082*** | 0.091*** | |
|  | (0.011) | (0.012) | (0.010) | (0.010) | |
| Wave 6 | 0.003 | 0.048*** | 0.009 | 0.063*** | |
|  | (0.006) | (0.005) | (0.006) | (0.005) | |
| AT | 0.064*** | -0.025 | 0.040** | 0.019 | |
|  | (0.020) | (0.018) | (0.020) | (0.014) | |
| SE | 0.175*** | 0.155*** | 0.202*** | 0.216*** | |
|  | (0.020) | (0.016) | (0.021) | (0.018) | |
| ES | -0.003 | -0.077*** | 0.060*** | 0.055*** | |
|  | (0.018) | (0.024) | (0.019) | (0.019) | |
| IT | 0.072*** | -0.101*** | 0.045** | -0.072*** | |
|  | (0.021) | (0.021) | (0.020) | (0.024) | |
| FR | 0.100*** | 0.036** | 0.067*** | 0.098*** | |
|  | (0.019) | (0.017) | (0.018) | (0.016) | |
| DK | 0.224*** | 0.124*** | 0.296*** | 0.185*** | |
|  | (0.016) | (0.017) | (0.018) | (0.020) | |
| CH | 0.173*** | 0.102*** | 0.113*** | 0.133*** | |
|  | (0.020) | (0.018) | (0.024) | (0.015) | |
| BE | 0.119*** | 0.097*** | 0.075*** | 0.104*** | |
|  | (0.018) | (0.015) | (0.018) | (0.015) | |
| CZ | 0.032* | -0.074*** | 0.078*** | 0.013 | |
|  | (0.017) | (0.014) | (0.016) | (0.017) | |
| SL | 0.055** | -0.144*** | 0.141*** | -0.067*** | |
|  | (0.022) | (0.023) | (0.020) | (0.018) | |
| ET | -0.084*** | -0.088*** | 0.029 | 0.039*** | |
|  | (0.017) | (0.017) | (0.018) | (0.012) | |
| IL | 0.088** | -0.131*** | 0.163*** | 0.038 | |
|  | (0.038) | (0.034) | (0.034) | (0.027) | |
| LU | 0.152*** | 0.065*** | 0.076*** | 0.116*** | |
|  | (0.023) | (0.020) | (0.023) | (0.020) | |
|  |  |  |  |  | |
| Observations | 19,188 | 19,188 | 22,681 | 22,681 | |
| Hansen p-value | 0.513 | 0.794 | 0.187 | 0.097 | |
| Weak identification | 132.141 | 132.141 | 217.888 | 217.888 | |

Note: Standard errors are clustered by country and cohort. *** p<0.01, ** p<0.05, * p<0.1. Stock-Yogo weak ID test critical values: 10% maximal IV size 19.93, 15% maximal IV size 11.59, 20% maximal IV size 8.75, 25% maximal IV size 7.25.

**Appendix B: Early and normal retirement eligibility criteria**

The initial sources of information about early and normal retirement eligibility criteria are Gruber and Wise (1999, 2010), Wise (2012), the Mutual Information System on Social Protection (MISSOC) database^[[1]](#footnote-1)^ and Social Security Administration (SSA) data on Social Security Programs throughout the World.^[[2]](#footnote-2)^ Other country specific auxiliary data sources are reported below. ER = early retirement. SR = statutory (normal) retirement.

Austria (see Staubli and Zweimüller, 2013)

ER: 60 for men and 55 for women until 2001. From 2001 until 2004, early retirement for men depends on year of birth. For men it is 61 for those born in 1942 and 62 for those born in 1943 onwards. For women from 2001 to 2010 the early retirement eligibility age is 56 for those born in 1947, 57 for those born between 1948 and 1951, 58 for those born between 1952 and 1954, 59 for those born in 1955 and 1956, 60 for those born in 1957 onwards. From 2005 onwards, the early retirement eligibility age is 62 for men.

SR: 65 for men and 60 for women.

Belgium (see Jousten *et* *al*., 2010)

ER: No early retirement until 1966, 60 afterwards for men, for women 55 until 1986 and 60 from 1987. For both men and women, 61 in 2014, 62 in 2016, but still possible to retire at age 60 with sufficient number of years of contributions.

SR: 65 for men, for women 60 until 1996, 61 from 1997 to 1999, 62 from 2000 to 2002, 63 from 2003 to 2005, 64 from 2006 to 2008, 65 from 2009.

Denmark (see Bingley *et* *al*., 2010)

ER: 60 for both men and women (Partial pension).

SR: 67 until 2003, 65 from 2004, for both men and women.

France (see Hamblin, 2013)

ER: No early retirement until 1963. 60 from 1963 to 1980, 55 from 1981 onwards.

SR: 65 until 1982 and 60 from 1983 to 2010; from 2011 60 for those born up to 1952, 61 for those born between 1953 and 1954 and 62 for those born since 1955.

Germany (see Berkel and Börsch-Supan, 2004, and Mazzonna and Peracchi., 2014, DRV 2015)

ER: For men, no early retirement until 1972, 60 from 1973 until 2005, 63 from 2006 onwards. For women, no early retirement in 1961, 60 from 1962.

SR: 65 for all until 2011, 67 from 2012.

Italy (see Angelini *et* *al*., 2009, and Mazzonna and Peracchi, 2014)

ER: from 1965 to 1995, early retirement was possible at any age with 35 years of contributions^[[3]](#footnote-3)^ (25 in the public sector) for both men and women; from 1996 it was stepwise increased up to 60 for both the private and public sector (61 for self-employed). From 2012 it is 62 for both men and women.

SR: The statutory retirement age was 60 (65 in the public sector) for men and 55 (60 in the public sector) for women from 1965 to 1993. Several consecutive reforms (1992, 1995 and 1998) increased the statutory retirement age to 65 for men and 60 for women with step-wise increments from 1994. The statutory retirement age is 66 from 2012 for men. For women, it is 65 in 2012 (there is the possibility to retire at 62 in the private sector and at 63 for self-employed), 66 in 2013 (63 for private sector employees and 64 for self-employed); in 2016 65 for private sector employees and 66 for self-employed.

Spain (see Blanco, 2000, and Mazzonna and Peracchi, 2014)

ER: 64 until 1982, 60 from 1983 to 1993, 61 from 1994 for both men and women. From 2013, 61 for involuntary early retirement or partial pension, 63 for voluntary early retirement, we take the lower.

SR: 65 for both men and women. Rising gradually to 67 from 2013 to 2027.

Sweden (see Mazzonna and Peracchi, 2014)

ER: No early retirement until 1962, 60 from 1963 to 1997, 61 from 1998 onwards.

SR: 67 for both men and women until 1994, 65 from 1995 onwards.

Switzerland (see Dorn and Sousa-Poza, 2003 and Mazzonna and Peracchi, 2014)

ER: No early retirement until 1996 for men and until 2000 for women. Then, 64 for men from 1997 until 2000 and 63 from 2001, for women 62 from 2001 (two years before SR age).

SR: 65 for men, for women 63 until 1963, 62 from 1964 until 2000, 63 from 2001 to 2004, 64 from 2005.

Czech Republic

ER: Early retirement is possible up to two years before normal retirement age.

SR: For men 60 from 1961 to 2002, 61 from 2003 to 2008, 62 from 2009 to 2015, 63 from 2016. For women, statutory retirement age depends on the number of children:

|  | 0 child | 1 child | 2 children | 3/4 children | 5+ children |
| --- | --- | --- | --- | --- | --- |
| Up to 1999 | 55 | 55 | 55 | 55 | 55 |
| From 2000 to 2002 | 56 | 56 | 56 | 56 | 56 |
| From 2003 to 2006 | 59 | 58 | 57 | 56 | 55 |
| From 2007 to 2011 | 60 | 59 | 58 | 57 | 56 |
| From 2012 to 2014 | 61 | 60 | 59 | 58 | 57 |
| From 2015 onwards | 62 | 61 | 60 | 59 | 58 |

Slovenia (OECD, 2013; Polanec et al, 2013; Guardiancich, 2010; Ahcan and Polanec, 2008; Majacen and Verbič, 2008; Mimir et al., 2004)

ER: Before 1993, no early retirement. From 1993 to 2014, 58 for male; 59 from 2015 onwards. For women, 53 from 1993 to 2001, 54 from 2002 to 2004, 55 from 2005 to 2007, 56 from 2008 to 2010, 57 in 2011 and 2013, 58 in 2014 and 2015, 59 from 2016 onwards.

SR: Before 1993, 55 for men 53 for women (40/38 years of contribution and no age requirements, we assume they start working at age 15). From 1993, 65 for men. For women, 61 from 2002 to 2004; 62 from 2005 to 2007; 63 from 2008 to 2013; 65 from 2014 onwards.

Estonia (Schmähl and Horstmann, 2002)

ER: 60 for men. For woman, 55 before 2004; 56 from 2004 to 2006; 57 from 2007 to 2009; 58 from 2010 to 2012; 59 from 2013 to 2015; 60 from 2016 onwards.

SR: 63 for men, for women 58 before 2004, 59 from 2004 to 2006, 60 from 2007 and 2009, 61 from 2010 to 2012, 62 from 2013 to 2015, 63 from 2016 onwards.

Israel

ER: No early retirement.

SR: 65 for men and 60 for woman up to 2004. From 2005 to 2009, 66 for men and 61 for women. 67 for men and 62 for women from 2009 onwards.

Luxemburg

ER: For both men and women, 65 as SR up to 1992. 60 from 1993 onwards.

SR: 65 for both men and women.

**Additional references**

Ahcan, A. and Polanec, S. (2008), ‘Social security and retirement during transition: Microeconometric evidence from Slovenia’, ENEPRI Research Report No. 58.

Angelini, V., Brugiavini, A. and Weber, G. (2009), ‘Ageing and unused capacity in Europe: Is there an early retirement trap?’, *Economic Policy*, vol. 24, pp. 463–508.

Berkel, B. and Börsch-Supan, A. (2004), ‘Pension reforms in Germany: The impact on retirement decisions’, MEA Discussion Paper 62.

Bingley, P., Datta Gupta, N. and Pedersen, P. J. (2010), ‘Social security, retirement and employment of the young in Denmark’, in J. Gruber and D. Wise (eds), *Social Security Programs and Retirement around the World. The Relationship to Youth Employment*, Chicago: University of Chicago.

Blanco, A. (2000), ‘The decision of early retirement in Spain’, FEDEA Working Paper no. 76.

Dorn, D., Sousa-Poza, A. (2003), ‘Why is the employment rate of older Swiss so high? An analysis of the social security system’, *The Geneva Papers on Risk and Insurance*, vol. 28, pp. 652–672.

DRV, 2015, Die richtige Altersrente für Sie. Available on line http://www.deutsche-rentenversicherung.de/Allgemein/de/Inhalt/5_Services/03_broschueren_und_mehr/01_broschueren/01_national/die_richtige_altersrente_fuer_sie.pdf?__blob=publicationFile&v=18 [last accessed on 25.01.2015]

Gruber, J. and Wise D. (1999), *Social Security and Retirement around the World*. Chicago: University of Chicago Press.

Gruber, J. and Wise D. (2010), *Social Security Programs and Retirement around the World: The Relationship to Youth Employment*. Chicago: University of Chicago Press.

Guardiancich, I. (2010), SLOVENIA Current pension system: first assessment of reform outcomes and output. European Social Security Observatory. Available online http://www.ose.be/files/publication/2010/country_reports_pension/OSE_2010_CRpension_Slovenia.pdf [last accessed on 29.01.2019]

Hamblin, K A. (2013), *Active Ageing in the European Union. Policy Convergence and Divergence*. London: Palgrave Macmillan.

Jousten, A., Lefèbvre, M., Perelman, S. and Pestieau, P. (2010), ‘The effects of early retirement on youth unemployment: The case of Belgium’, in J. Gruber and D. Wise (eds), *Social Security Programs and Retirement around the World. The Relationship to Youth Employment*, Chicago: University of Chicago Press.

Majcen, B. and Verbič, M. (2008), ‘Slovenian pension system in the context of upcoming demographic developments’, in R. Hozmann, L. MacKellar and J. Repansek (eds.), *Pension Reform in South-Eastern Europe: Linking to Labor and Financial Market Reforms,* Washington DC: World Bank.

Mrak, Mojmir [editor]; Rojec, Matija [editor]; Silva-Jauregui, Carlos [editor]; Drnovsek, Janez; Gligorov, Vladimir; Bucar, Bojko; Borak, Neven; Borak, Bistra; Mencinger, Joze; Rant, Andrej; Arhar, France; Marjcen, Boris; Kaminski, Bartlomiej; Ribnikar, Ivan; Kosak, Tomaz; Bole, Velimir; Cvikl, Milan M.; Gaspari, Mitja; Ovin, Rasto; Kramberger, Bostjan; Simoneti, Marko; Gregoric, Aleksandra; Domadenik, Polona; Prasnikar, Janez; Stiblar, Franjo; Voljc, Marko; Mramor, Dusan; Jasovic, Bozo; Vodopivec, Milan; Stanovnik, Tine; Damijan, Joze; Inotai, Andras; Stanovinik, Peter; Potcnik, Janez; Lombardero, Jaime Garcia; Sabic, Zlatko; Svetlicic, Marjan; Pyszna-Nigge, Dorota; Wessels, Wolfgang; Sustersic, Janez. 2004. Slovenia: from Yugoslavia to the European Union (English). Washington, DC: World Bank. Available on line: <http://documents.worldbank.org/curated/en/197621468776951986/Slovenia-from-Yugoslavia-to-the-European-Union> [last accessed on 29.01.2019]

Mazzonna, F. and Peracchi, F. (2014), ‘Unhealthy retirement?’, EIEF Working Paper 09/14.

OECD (2013), ‘Slovenia’, in *Pensions at a Glance 2013: OECD and G20 Indicators*, Paris: OECD Publishing.

Polanec, S., Ahčan, A. and Verbič, M. (2013), ‘Retirement decisions in transition: Microeconometric evidence from Slovenia’, *Post-Communist Economies*, vol. 25, pp. 99-118

Schmähl, W. and Horstmann, S. (2002), *Transformation of Pension Systems in Central and Eastern Europe*, Edward Elgar.

Staubli, S. and Zweimüller, J. (2013), ‘Does raising the retirement age increase employment of older workers?’, *Journal of Public Economics*, vol. 108, pp. 17-32.

Wise, D. (2012), *Social Security Programs and Retirement around the World: Historical Trends in Mortality and Health, Employment, and Disability Insurance Participation and Reforms*. Chicago: University of Chicago Press.

**Appendix C: Retirement decisions and age eligibility**

Figure C1: Proportion of retired since/to statutory retirement age. All countries.

Figure C2: Proportion of retired since/to early retirement age. All countries.

Figure C3: Proportion of retired since/to statutory retirement age by country. Males.

Figure C4: Proportion of retired since/to statutory retirement age by country. Females.

Figure C5: Proportion of retired since/to early retirement age by country. Males.

Figure C6: Proportion of retired since/to early retirement age by country. Females.

**Appendix D: Robustness analyses – additional tables**

Table D1: Probability of having at least very good computer skills. Linear probability models estimated by OLS and 2SLS for men and women.

|  | Men | | Women | |
| --- | --- | --- | --- | --- |
|  | (1) | (2) | (3) | (4) |
| VARIABLES | OLS | 2SLS | OLS | 2SLS |
|  |  |  |  |  |
| Retired | -0.039*** | -0.032 | -0.062*** | -0.052** |
|  | (0.010) | (0.038) | (0.009) | (0.021) |
|  |  |  |  |  |
| Observations | 19,188 | 19,188 | 22,681 | 22,681 |
|  |  |  |  |  |
|  |  |  |  |  |
| Sargan-Hansen p-value |  | 0.483 |  | 0.930 |

Note: Additional controls: couple, education dummies, age, age squared, poor health, adl, iadl, household wealth quartiles, blue collar, public sector, have children, have grandchildren, time dummy, country dummies. Standard errors are clustered at the individual level for OLS estimates and by country and cohort for 2SLS estimates. *** p<0.01, ** p<0.05, * p<0.1. Stock-Yogo weak ID test critical values: 10% maximal IV size 19.93, 15% maximal IV size 11.59, 20% maximal IV size 8.75, 25% maximal IV size 7.25.

1. https://www.missoc.org/missoc-database/comparative-tables/ [↑](#footnote-ref-1)
2. https://www.ssa.gov/policy/docs/progdesc/ssptw/ [↑](#footnote-ref-2)
3. We use work experience to define eligibility. [↑](#footnote-ref-3)
